# Supplementary material for: Metabolic engineering of doxorubicin biosynthesis through P450-redox partner optimization and structural analysis of DoxA
Source: Nat Commun. 2026 Feb 4;17:2358. doi: 10.1038/s41467-026-69194-6 (PMC12979805; doi:10.1038/s41467-026-69194-6)

Supplementary Information for:

Metabolic Engineering of Doxorubicin Biosynthesis through P450-Redox Partner Optimization and Structural Analysis of DoxA

Authors:

Arina Koroleva[1,§], Erika Artukka[1,§], Keith Yamada[1,§], Sean A. Newmister[2,§], Ralph J. Harte[2], Hannah Boesger[2], Mikael Londen[1], Jacob N. Sanders[3], Heli Tirkkonen[1], Matti Kannisto[1], Rosan C. M. Kuin[4], Mandy Hulst[5], Rongbin Wang[1], Ester Leskinen[1], Morgane Barillec[1], Jarmo Niemi[1], Gilles P. van Wezel[5], Jacques Neefjes[4], S. Eric Nybo[6], Kendall N. Houk[3], David H. Sherman[2], Robbert Q. Kim[4] and Mikko Metsä-Ketelä[1,*]

Affiliations:

[1]Department of Life Technologies, University of Turku, FIN-20014 Turku, Finland

[2]Life Sciences Institute, University of Michigan, Ann Arbor, Michigan 48109, USA

[3]Department of Chemistry, University of California, Los Angeles, 90095, California, USA

[4]Department of Cell and Chemical Biology, Leiden University Medical Centre, Leiden, The Netherlands

[5]Institute of Biology, Leiden University, Sylviusweg 72, 2333 BE Leiden, The Netherlands

[6]Department of Pharmaceutical Sciences, College of Pharmacy, Ferris State University, Big Rapids, Michigan 49307, USA

[§] Equal contribution

* Corresponding author E-mail: mianme@utu.fi

Contents

Supplementary Table 1. Comparative transcriptomics analysis of the daunorubicin biosynthetic gene cluster and ferredoxin clusters in *S. peucetius* ATCC 27952, GOO1 and X121 strains.

Supplementary Table 2. NMR data for 7-deoxydaunoribicinone

Supplementary Table 3. Crystallographic data collection and refinement statistics.

Supplementary Table 4. Optimized quantum mechanical geometries

Supplementary Table 5. Statistics of ligand productive poses in MD simulations

Supplementary Table 6. NMR data for ε-rhodomycinone

Supplementary Table 7. The sequence of oligonucleotides for RT-qPCR

Supplementary Figure 1. Fold-change in mRNA expression of the *fdx4* gene in *Streptomyces peucetius* ATCC 27952 and its Δ*sufR* mutant.

Supplementary Figure 2. Analysis of proteins used in this study by SDS-PAGE.

Supplementary Figure 3. Spectroscopic analysis of proteins used in this study.

Supplementary Figure 4. Reductive deglycosylation of anthracyclines by ferredoxin reductases.

Supplementary Figure 5. ^{1}H NMR spectrum of 7-deoxydaunorubicinone

Supplementary Figure 6. ^{13}C NMR spectrum of 7-deoxydaunorubicinone

Supplementary Figure 7. COSY NMR spectrum of 7-deoxydaunorubicinone

Supplementary Figure 8. HMBC NMR spectrum of 7-deoxydaunorubicinone

Supplementary Figure 9. HSQC NMR spectrum of 7-deoxydaunorubicinone

Supplementary Figure 10. HR-MS spectrum of 7-deoxydaunorubicinone

Supplementary Figure 11. Identification of SpFdx4 as a Rieske-type ferredoxin.

Supplementary Figure 12. MRD does not interact with anthracyclines or prevent reductive deglycosylation.

Supplementary Figure 13. Structural and functional analysis of DoxA.

Supplementary Figure 14. Molecular dynamics simulations of DoxA with DOD and DNR.

Supplementary Figure 15. Interaction of amino acid residue with ligands in MD simulations.

Supplementary Figure 16. ^{1}H NMR spectrum of ε-rhodomycinone.

Supplementary Figure 17. ^{13}C NMR spectrum of ε-rhodomycinone.

Supplementary Figure 18. HR-MS spectrum of ε-rhodomycinone.

Supplementary Table 1. Comparative transcriptomics analysis of the daunorubicin biosynthetic gene cluster and ferredoxin clusters in *S. peucetius* ATCC 27952, GOO1 and X121 strains.

Name	Annotation	Accession number	WT vs GOO1						WT vs X121						GOO1 vs X121					
			Day 1	Day 2	Day 3	Day 4	Day 5	Day 6	Day 1	Day 2	Day 3	Day 4	Day 5	Day 6	Day 1	Day 2	Day 3	Day 4	Day 5	Day 6
**Dnr cluster**																				
drrC	ABC transporter	fig|316280.9.peg.5137	1,2	2,6	3,0	4,2	2,5	-1,0	-1,7	0,0	0,0	-0,6	-2,6	-4,4	-2,8	-2,2	-3,6	-4,7	-5,0	-3,4
???	Mobile element protein	fig|316280.9.peg.5138	0,0	5,1	3,7	4,6	2,3	0,0	0,0	0,0	0,0	0,0	0,0	-4,4	0,0	0,0	-4,1	-5,6	-3,4	-3,6
dnrS	hypothetical protein	fig|316280.9.peg.5139	10,1	11,5	6,5	4,3	0,4	1,4	7,0	7,0	3,7	1,4	0,0	1,5	-2,9	-4,1	-2,8	-2,8	-0,5	0,0
dnrQ	hypothetical protein	fig|316280.9.peg.5140	7,8	11,3	7,7	4,2	0,5	1,6	5,0	7,3	5,0	1,5	0,0	2,1	-2,7	-3,6	-2,6	-2,6	-0,9	0,5
dnrP	Alpha/beta hydrolase fold	fig|316280.9.peg.5141	6,8	8,8	9,0	4,9	0,6	1,6	3,6	6,0	6,6	2,2	0,0	2,2	-3,2	-2,7	-2,4	-2,6	-0,7	0,6
dnrK	hypothetical protein	fig|316280.9.peg.5142	11,9	13,1	9,2	5,1	0,6	1,6	8,5	9,3	6,7	2,4	0,0	2,1	-3,1	-3,3	-2,4	-2,6	-0,7	0,5
dnrD	nogalonic acid methyl ester cyclase	fig|316280.9.peg.5143	11,3	9,7	8,5	4,0	0,6	1,5	8,2	6,4	6,1	1,5	0,0	1,8	-2,8	-3,2	-2,3	-2,4	-0,7	0,3
dnrC	ORF5	fig|316280.9.peg.5144	6,7	10,8	11,0	4,9	0,4	1,1	4,7	8,2	9,6	3,2	0,4	1,8	-1,9	-2,2	-1,3	-1,6	0,0	0,8
dpsD	hypothetical protein	fig|316280.9.peg.5145	4,6	9,2	9,3	3,6	-1,0	2,3	2,9	6,7	7,3	0,0	0,0	2,0	-1,6	-2,2	-1,9	-2,2	0,0	0,0
dpsC	putative 3-oxoacyl-ACP synthase III	fig|316280.9.peg.5146	9,3	10,4	7,3	4,3	-0,7	2,6	6,5	7,5	5,2	1,8	0,0	2,4	-2,6	-2,5	-2,1	-2,4	0,0	0,0
dpsB	Ketosynthase, beta subunit	fig|316280.9.peg.5147	7,1	6,9	7,6	3,4	0,0	2,3	4,8	4,2	5,7	1,2	0,0	2,1	-2,2	-2,5	-1,9	-2,1	0,0	0,0
dpsA	Ketosynthase, alpha subunit	fig|316280.9.peg.5148	8,0	8,6	11,6	3,8	-0,5	2,3	5,5	5,9	9,5	1,5	0,0	2,5	-2,5	-2,6	-1,9	-2,2	0,0	0,0
dnrG	anthrone oxygenase	fig|316280.9.peg.5149	9,2	10,4	10,6	3,8	0,0	2,0	6,7	7,9	8,5	2,0	0,0	2,2	-2,3	-2,2	-1,9	-1,7	0,0	0,0
dpsE	acyl-carrier protein reductase	fig|316280.9.peg.5150	6,7	11,5	8,8	4,5	0,0	1,8	4,9	8,7	6,8	2,4	0,0	1,9	-1,8	-2,4	-2,0	-2,0	0,0	0,0
dpsF	hypothetical protein	fig|316280.9.peg.5151	10,1	11,7	11,9	3,9	0,0	1,3	7,8	8,6	9,6	1,8	0,0	1,3	-2,1	-2,7	-2,2	-2,0	0,0	0,0
dauE	hypothetical protein	fig|316280.9.peg.5152	9,5	7,0	10,8	3,2	0,0	0,8	7,5	5,9	10,0	2,5	0,8	1,1	-1,8	-1,0	-0,7	-0,6	0,7	0,3
dnrH	hypothetical protein	fig|316280.9.peg.5153	8,6	9,8	6,5	2,1	-2,2	3,5	6,4	6,9	5,0	0,0	0,0	3,3	-2,0	-2,5	-1,5	-1,1	1,9	0,0
dnmT	hypothetical protein	fig|316280.9.peg.5154	10,1	8,2	7,8	3,1	-1,9	3,4	7,7	5,7	6,5	1,7	-0,9	3,2	-2,2	-2,4	-1,3	-1,2	1,0	0,0
dpsH	hydroxylacyl-CoA dehydrogenase	fig|316280.9.peg.5155	5,8	9,5	8,7	1,9	-2,2	3,0	4,0	7,0	7,6	0,0	-0,8	2,7	-1,8	-2,1	-1,0	0,0	1,5	0,0
dpsG	hypothetical protein	fig|316280.9.peg.5156	10,1	7,7	11,5	2,6	-0,7	2,8	7,7	4,7	9,2	0,0	-0,5	2,6	-2,1	-2,9	-2,2	-1,8	0,0	0,0
dnrU	hypothetical protein	fig|316280.9.peg.5157	10,6	11,8	11,7	4,0	-0,6	2,6	8,0	8,5	9,2	1,8	-0,4	2,7	-2,3	-2,9	-2,4	-2,0	0,0	0,0
dnrV	Putative hydroxylase	fig|316280.9.peg.5158	11,5	12,5	9,1	3,7	-0,9	3,0	8,7	8,8	6,6	1,4	-0,8	2,9	-2,5	-3,3	-2,4	-2,3	0,0	0,0
doxA	P450 mono-oxygenase	fig|316280.9.peg.5159	9,3	12,6	8,5	3,8	-0,7	2,7	6,4	8,9	5,8	1,2	-0,7	2,6	-2,8	-3,4	-2,7	-2,5	0,0	0,0
dnrI	Regulatory protein	fig|316280.9.peg.5160	7,9	8,4	9,1	4,1	2,2	-0,6	6,5	8,5	9,6	4,6	3,7	1,7	-1,3	0,2	0,5	0,5	1,5	2,3
dnrJ	Aminotransferase	fig|316280.9.peg.5161	7,6	7,8	8,8	4,5	2,3	-0,5	6,5	8,0	9,0	4,7	3,4	1,6	-1,1	0,4	0,2	0,3	1,1	2,1
dnmV	hypothetical protein	fig|316280.9.peg.5162	6,1	10,1	6,3	2,0	-1,3	4,1	0,0	5,3	3,3	0,0	-1,2	3,4	-4,1	-4,4	-3,0	-1,9	0,0	-0,7
dnmU	dTDP-glucose epimerase	fig|316280.9.peg.5163	8,7	9,9	5,7	2,6	-1,4	2,5	0,0	5,5	3,2	0,0	-1,0	2,1	-4,0	-4,0	-2,4	-2,0	0,0	0,0
???	Acyl-CoA dehydrogenase	fig|316280.9.peg.5164	7,8	11,7	11,4	2,1	-1,5	3,0	4,9	7,3	8,8	0,0	-1,3	2,9	-2,8	-4,0	-2,5	-1,4	0,0	0,0
???	hypothetical protein	fig|316280.9.peg.5165	0,0	0,0	0,0	0,0	0,0	0,0	0,0	0,0	0,0	0,0	0,0	0,0	0,0	0,0	0,0	0,0	0,0	0,0
dpsY	FIG00816365: hypothetical protein	fig|316280.9.peg.5166	10,4	11,5	11,1	2,7	-2,0	3,3	7,9	8,5	9,7	2,3	-0,5	3,5	-2,3	-2,6	-1,3	0,0	1,5	0,0
dnrX	C-methyltransferase	fig|316280.9.peg.5167	7,1	11,8	6,8	2,5	-1,9	2,9	4,5	8,5	5,0	1,5	-0,4	3,1	-2,5	-2,9	-1,8	-0,8	1,6	0,3
???	FIG00817184: hypothetical protein	fig|316280.9.peg.5168	2,9	8,1	6,1	4,5	2,0	0,0	0,0	0,0	0,0	0,0	-1,7	-1,7	-3,4	-3,1	-3,5	-4,4	-3,6	-1,1
dnrW	Putative oxidoreductase	fig|316280.9.peg.5169	4,0	5,3	4,5	5,7	2,4	-0,8	0,0	2,0	0,0	0,0	-1,5	-2,5	-3,5	-3,2	-3,8	-5,1	-3,8	-1,7
drrB	ABC transporter, permease protein	fig|316280.9.peg.5170	3,7	4,1	4,5	4,9	2,6	-1,0	0,0	0,0	0,0	0,0	-2,2	-3,8	-5,7	-3,8	-4,8	-5,2	-4,8	-2,9
drrA	ABC transporter, ATP-binding protein	fig|316280.9.peg.5171	3,3	5,4	4,8	5,2	2,6	-1,2	0,0	1,8	0,0	0,0	-2,2	-3,7	-3,9	-3,4	-4,7	-5,3	-4,8	-2,5
dnrF	hypothetical protein	fig|316280.9.peg.5172	7,9	10,9	6,1	2,2	-1,5	2,7	5,3	7,7	4,7	1,0	-0,7	2,5	-2,5	-2,8	-1,4	-1,0	0,8	0,0
dnrO	Transcriptional regulator	fig|316280.9.peg.5173	0,0	0,0	0,8	1,8	1,1	-0,6	0,0	0,0	0,0	0,0	-0,7	-2,3	0,0	0,0	-1,3	-1,6	-1,8	-1,7
dnrN	Regulatory protein	fig|316280.9.peg.5174	3,4	2,1	2,4	2,1	0,0	0,0	2,6	1,7	1,3	0,0	-0,7	0,0	0,0	0,0	-1,1	-1,3	-1,1	0,0
dnmM	dTDP-glucose dehydratase	fig|316280.9.peg.5175	4,3	9,0	5,7	3,1	0,0	1,5	2,5	5,2	0,0	0,0	0,0	0,0	-1,8	-3,4	-3,0	-2,3	0,0	-0,9
dnmL	Thymidylyltransferase	fig|316280.9.peg.5177	8,5	7,4	10,1	3,5	-0,9	2,7	6,0	4,4	7,7	1,6	-1,0	2,1	-2,3	-2,9	-2,3	-1,9	0,0	-0,6
**Suf cluster**																				
	VOC family protein	WP_100106552.1	0,0	0,0	5,5	0,0	0,0	0,0	0,0	0,0	0,0	0,0	0,0	0,0	3,8	2,1	0,0	0,0	0,0	0,0
sufT	Fe-S cluster assembly protein	WP_100106553.1	-1,2	1,0	2,6	0,7	2,0	-2,4	1,9	2,4	3,2	1,1	2,3	0,0	3,1	1,5	0,6	0,5	0,0	2,3
sufU	Zinc-dependent sulfurtransferase	WP_100106554.1	-0,9	1,5	2,2	2,2	1,7	-1,3	2,3	3,2	2,9	2,8	2,0	0,0	3,2	1,8	0,8	0,7	0,3	1,5
sufS	Cysteine desulfurase	WP_100106555.1	0,0	1,2	2,0	2,5	2,3	-1,7	3,0	3,6	3,0	3,4	2,7	0,7	3,5	2,5	1,1	1,0	0,5	2,4
sufC	Fe-S cluster assembly ATPase	WP_100106556.1	0,0	1,1	2,1	2,3	2,5	-1,9	2,5	3,3	3,1	3,2	2,8	0,7	2,7	2,3	1,0	1,0	0,4	2,6
fdx4	Ferredoxin 2Fe-2S	WP_100106557.1	0,0	1,6	1,8	2,5	2,4	-1,6	3,4	3,9	2,4	3,8	2,7	0,8	3,0	2,4	0,7	1,4	0,0	2,5
sufD	Fe-S cluster assembly protein	WP_100106558.1	0,0	1,4	2,1	2,2	2,1	-1,4	2,6	3,7	3,0	3,2	2,6	0,9	3,2	2,5	1,0	1,1	0,5	2,2
sufB	Fe-S cluster assembly protein	WP_100106559.1	0,0	1,6	2,1	2,8	2,0	-1,5	2,8	3,9	3,3	3,9	2,4	0,7	3,1	2,4	1,2	1,3	0,4	2,2
sufR	HTH-type transcriptional repressor	WP_100106560.1	-0,5	0,7	1,7	1,8	2,0	-1,8	2,6	2,7	2,7	3,1	2,8	0,4	3,2	2,0	1,0	1,4	0,8	2,2
**Tau cluster**																				

ssuA	Aliphatic sulfonate-binding protein	ATW52908.1	0,0	0,0	0,0	0,0	0,0	0,0	8,8	12,1	10,1	7,8	8,7	11,0	9,0	8,8	10,7	7,0	12,1	8,0
tauD	Taurine dioxygenase	WP_100109757.1	0,0	0,0	0,0	1,8	0,0	0,0	7,1	9,1	8,9	8,8	9,5	8,0	10,3	14,1	8,2	7,2	9,0	9,4
fdx5	Ferredoxin 4Fe-4S	WP_100109758.1	0,0	0,0	0,0	0,0	0,0	0,0	7,8	8,5	9,7	11,4	11,1	10,0	7,9	11,7	10,3	6,8	7,8	9,8
sdhA	Succinate dehydrogenase/reductase	WP_100109759.1	0,0	0,0	0,0	0,0	0,0	0,0	5,3	8,6	6,8	6,7	9,1	6,0	6,4	8,9	10,5	6,6	8,2	10,2
ssuD	Alkanesulfonate monooxygenase	WP_100109760.1	0,0	0,0	0,0	0,0	0,0	0,0	6,8	12,0	10,8	8,4	9,9	11,5	7,0	10,5	9,2	6,9	8,0	6,6
tauC	ABC transporter permease protein	WP_100109761.1	0,0	0,0	0,0	0,0	0,0	0,0	7,6	10,5	9,4	7,8	10,6	10,0	7,7	11,0	10,0	7,0	8,7	9,8
tauB	ABC transporter ATP-binding protein	WP_100109762.1	0,0	0,0	0,0	0,0	0,0	0,0	7,4	10,5	9,3	6,4	10,7	9,7	7,5	11,1	9,9	6,0	11,0	9,6
tauA	ABC transporter	WP_100109763.1	0,0	0,0	0,0	0,0	0,0	0,0	9,1	12,3	11,2	9,8	12,5	7,5	9,2	10,8	9,5	7,3	10,6	8,5
sfnB	Sulfur acquisition oxidoreductase	WP_100109765.1	0,0	0,0	0,0	2,4	0,0	0,0	7,7	12,5	11,7	9,2	10,9	12,3	10,9	13,1	10,0	6,9	11,3	8,4
glnR	Regulatory protein	WP_100109766.1	0,0	0,0	0,0	4,3	0,0	0,0	9,4	13,6	10,2	11,4	10,8	10,2	9,7	12,1	13,8	7,2	12,5	10,7

Supplementary Table 2. NMR data for 7-deoxydaunorubicinone

	^{13}C	^{1}H	HMBC	COSY
**Position**	**δ ppm**	**δ ppm, *J* Hz**		
1	119.7	7.99 (dd, 1.1, 7.7)	3, 4 (weak), 4a, 12, 12a (weak)	2
2	135.6	7.75 (dd, 7.7, 8.4)	1 (weak), 3 (weak), 4, 12a	1, 3
3	118.2	7.36 (dd, 0.6, 8.4)	1, 2 (weak), 4, 5 (weak)	2
4	161.1			
4-OMe	56.8	4.08 (s)	3 (weak), 4	
4a	121.2			
5	187.2			
5a	110.6			
6	156.3			
6-OH		13.80 (s)	5a, 6, 6a	
6a	138.3			
7	20.0	3.13 (ddd, 18.0, 5.8, 1.6), 2.92 (m, overlap with H-10)	2.92: 6a, 8, 9, 10a; 3.13: 6, 6a, 8, 9, 10a	3.13: 7 (2.92), 8 (2.01); 2.92: 7 (3.13), 8 (2.01)
8	29.2	2.01 (td, 12.5, 5.8), 1.94 (ddt, 12.5, 6.3, 2.1)	1.94: 6a, 7, 9, 10; 2.01: 7, 13	1.94: 7 (2.92, 3.13), 8 (2.01), 10 (2.90); 2.01: 7 (2.92, 3.13), 8 (1.94)
9	75.9			
9-OH		3.80 (s)	8, 9, 10, 13	
10	32.5	3.03 (dd, 1.9, 18.0), 2.90 (dd, 2.2, 18.0)	6a, 8, 9, 10a, 11, 13	3.03: 10 (2.90); 2.90: 10 (3.03)
10a	133.2			
11	156.4			
11-OH		13.39 (s)	10a, 11, 11a	
11a	109.8			
12	186.4			
12a	135.9			
13	211.4			
14	24.0	2.39 (s)	9, 13	

Supplementary Table 3. Data collection and refinement statistics

	DoxA-DOD (**9S7F**)	DoxA-DHD (**9SI5**)	DoxA-DNR (**9O35**)
**Data collection**			
Space group	$C222_1$	$C222_1$	$C222_1$
Cell dimensions			
a, *b*, *c* (Å)	101.8, 107.7, 184.7	101.9, 108.1, 179.8	101.8, 108.9, 182.0
α, β, γ (°)	90, 90, 90	90, 90, 90	90, 90, 90
Resolution (Å)	73.97 - 1.77 (1.80-1.77)	89.89 – 2.54 (2.65-2.54)	46.74 - 2.194 (2.25 - 2.19)
R_{pim}	0.043 (0.728)	0.138 – (1.088)	0.041 (0.490)
$I / \sigma I$	9.3 (1.2)	2.9 (0.2)	11.70 (1.28)
Completeness (%)	99.0 (94.6)	99.4 (95.0)	98.98 (89.95)
Redundancy	5.6 (5.6)	6.6 (5.3)	6.9 (4.2)
$CC_{1/2}$	0.986 (0.278)	0.874 (0.472)	0.998 (0.739)
**Refinement**			
Resolution (Å)	73.0 – 1.77	89.0 – 2.54	47.0 - 2.19
No. reflections	97802 (4814)	32714 (1640)	51489 (3275)
R_{work} / R_{free}	0.194 / 0.222	0.233 / 0.281	0.209 / 0.257
No. atoms	6775	6585	6678
Protein	6273	6287	6204
Ligand/ion	160	162	167
Water	342	136	307
B-factors	38.0	70	37.77
Protein	39.3	70.9	38.03
Ligand/ion	24.8	54.6	29.99
Water	39.7	56.5	36.72
R.m.s. deviations			
Bond lengths (Å)	0.015	0.0125	0.009
Bond angles (°)	2.325	2.331	1.02

*Values in parentheses are for highest-resolution shell.

Supplementary Table 4. Optimized quantum mechanical geometries

DOD optimization

```
C                   4.39706900    1.36988900    1.10599700
O                   3.50541600    0.96829200    0.08209900
C                   3.11634300   -0.39629100    0.14134700
O                   1.92082500   -0.54246200   -0.57780600
C                   0.74931800    0.11350800   -0.01777300
C                   0.48008300    1.41450400   -0.77786900
C                  -0.92557400    1.97901000   -0.51402300
C                  -1.09211200    2.41210400    0.96195300
C                  -2.47054700    2.97787400    1.32041700
O                  -1.16313000    3.10164000   -1.37758400
C                  -1.94617100    0.92176300   -0.94816300
C                  -0.42303500   -0.85305900   -0.03703100
C                  -1.65596900   -0.45782200   -0.39762600
C                  -2.85887600   -1.38162800   -0.29765300
O                  -3.94323800   -0.74552400    0.41459000
C                  -0.11639300   -2.25720700    0.45238000
O                   0.65861800   -2.99653000   -0.50577200
C                  -1.34494600   -3.07282500    0.73074900
C                  -2.57111400   -2.68126800    0.39404800
C                   4.14341700   -1.31812700   -0.49952800
H                   4.52390600    2.45053100    1.00627800
H                   3.99350600    1.14780300    2.10657100
H                   5.38325300    0.89189000    1.01492000
H                   2.95339100   -0.66330200    1.20303700
H                   0.98579500    0.33373600    1.03487900
H                   1.25532700    2.14575200   -0.52152600
H                   0.57006400    1.20857700   -1.85050500
H                  -0.86798600    1.56444400    1.62071200
H                  -0.31854100    3.16885200    1.16641600
```

H	-2.45126600	3.43515900	2.31591900
H	-2.77824800	3.74084100	0.59822100
H	-3.23146700	2.19038300	1.33766800
H	-0.51597600	3.78699900	-1.14322400
H	-2.95149800	1.23983100	-0.66066500
H	-1.93641400	0.88878800	-2.04758600
H	-3.20377100	-1.60769700	-1.32256000
H	-4.50803200	-0.30327200	-0.23545300
H	0.46473300	-2.17058800	1.39118900
H	1.34087200	-2.37175400	-0.81081500
H	-1.17589400	-4.04361900	1.19113500
H	-3.44048300	-3.30196900	0.59759300
H	4.26354900	-1.05015000	-1.55317800
H	5.11215700	-1.22680800	0.00137500
H	3.81615300	-2.36030800	-0.42796200

DOD radical optimization

C	4.39573300	1.26277700	1.14664300
O	3.51436100	0.88687500	0.10441400
C	3.07825400	-0.46385000	0.15985200
O	1.89583700	-0.57246100	-0.58651900
C	0.73623800	0.12700600	-0.05686600
C	0.51614000	1.42069900	-0.84356600
C	-0.85567800	2.03727000	-0.53854000
C	-0.96108300	2.45843400	0.90201000
C	-2.26626100	2.72132400	1.57807200
O	-1.08482700	3.18659700	-1.39797100
C	-1.93545300	1.03090000	-0.95276200
C	-0.46698200	-0.80172400	-0.07116800
C	-1.68992700	-0.36148200	-0.41230900
C	-2.92632300	-1.23507000	-0.28643500
O	-3.95331200	-0.57013100	0.48562600
C	-0.20417600	-2.21737900	0.41033500
O	0.55072100	-2.97481400	-0.54931800
C	-1.45616500	-2.99881100	0.68046900
C	-2.67259000	-2.56049100	0.36726700
C	4.08833000	-1.42265300	-0.45310400
H	4.56087700	2.33819400	1.04614100
H	3.96281400	1.05827600	2.13871200
H	5.36726300	0.75192400	1.07951800
H	2.88168400	-0.72001000	1.21847800
H	0.95887600	0.36507800	0.99463900
H	1.32592900	2.12209600	-0.61333900
H	0.57137400	1.19561000	-1.91432600
H	-0.03702400	2.72930500	1.41123300
H	-2.85859100	1.80050600	1.69308400
H	-2.12276500	3.15324400	2.57327600
H	-2.88599300	3.41981300	0.99366700
H	-0.46461100	3.87770400	-1.11218400
H	-2.91553500	1.39694500	-0.63427700
H	-1.95844200	1.00575600	-2.05200200
H	-3.32469100	-1.42097500	-1.29985800
H	-4.49992100	-0.05568600	-0.12609900
H	0.37571400	-2.15347700	1.35183500
H	1.25886300	-2.37301000	-0.84133300
H	-1.31325900	-3.98526100	1.11579000
H	-3.55889300	-3.15781100	0.56779900
H	4.24292100	-1.16294700	-1.50434100
H	5.04730700	-1.36286800	0.07082300
H	3.72351900	-2.45262800	-0.38648000

Transition state for DOD H-atom transfer with CpdI

C	1.95656100	4.26696100	0.46118300
O	2.65782300	3.42351000	-0.43108000
C	3.95506900	3.05320600	0.00025100
O	4.32425300	1.87799500	-0.66916300
C	3.64253800	0.65463300	-0.27080100
C	2.46699700	0.37838800	-1.21402600
C	1.95480500	-1.07373800	-1.14740600
C	1.33194100	-1.40714300	0.21930600

C	0.96505800	-2.84783700	0.48469200
O	0.98325500	-1.29392500	-2.17078300
C	3.13559600	-1.99918400	-1.48818400
C	4.66195800	-0.47277400	-0.20183100
C	4.38657300	-1.69466400	-0.69302400
C	5.29968700	-2.88492700	-0.46706900
O	4.58248800	-3.95203600	0.19795000
C	5.95403400	-0.15264000	0.53325900
O	6.76695300	0.78812600	-0.18241100
C	6.82609400	-1.35281700	0.76959300
C	6.55242300	-2.56691700	0.29436100
C	4.99670500	4.11163000	-0.33933500
H	0.94389800	4.37044500	0.06561900
H	1.89980000	3.83023300	1.47131800
H	2.41216600	5.26603800	0.53942200
H	3.92157900	2.86919000	1.09149900
H	3.27315700	0.82703300	0.75391700
H	1.65719300	1.08217900	-1.00635400
H	2.80616600	0.56067100	-2.23980900
H	1.82000100	-0.91800000	1.06846100
H	0.16399800	-0.69993100	0.13941400
H	0.35669600	-2.94475800	1.38829600
H	0.41189900	-3.27095500	-0.35913200
H	1.87113500	-3.45645000	0.62691800
H	0.20031300	-0.77003000	-1.90342100
H	2.85549900	-3.04726600	-1.36031700
H	3.34307600	-1.86351300	-2.55989700
H	5.55714100	-3.32832000	-1.43903200
H	4.43454000	-3.64807600	1.10901100
H	5.68479200	0.28330700	1.51648600
H	6.13699200	1.42732600	-0.56266400
H	7.74858200	-1.15898500	1.31304400
H	7.22475100	-3.40466600	0.46891300
H	5.02862800	4.25275600	-1.42375300
H	4.74606000	5.06670100	0.13305600
H	5.98566600	3.80036400	0.01252400
Fe	-2.34156800	0.09163600	0.16000600
N	-1.92572800	0.22732500	2.13984300
N	-2.56939800	-1.91463200	0.39494600
N	-2.30548100	2.09751300	-0.00836100
N	-2.93572500	-0.05000400	-1.75011500
C	-1.62551500	1.37912100	2.82138200
C	-2.86427800	-2.81641000	-0.59576300
C	-1.81985300	-0.79244300	3.05134200
C	-2.41291700	-2.65358700	1.54821500
C	-1.96852800	2.98883900	0.97357500
C	-3.14424200	-1.20860600	-2.45584300
C	-2.56175500	2.84131700	-1.12707600
C	-3.09288900	0.97644000	-2.64999700
C	-1.31362300	1.07683500	4.19774900
C	-2.90357900	-4.15573000	-0.05623600
C	-1.43561600	-0.27189500	4.34066300
C	-2.62965000	-4.05298000	1.27292500
C	-2.01462700	4.33970900	0.45955800
C	-3.44871400	-0.90506200	-3.83413900
C	-2.38360000	4.24756500	-0.84738700
C	-3.42286000	0.45047300	-3.95295500
H	-1.04680500	1.81264200	4.94579300
H	-3.11450900	-5.04664900	-0.63400600
H	-1.28842900	-0.87238300	5.22932100
H	-2.56715900	-4.84259000	2.01082900
H	-1.80042500	5.22866100	1.03951100
H	-3.65204100	-1.64532500	-4.59738800
H	-2.52910700	5.04357400	-1.56646200
H	-3.59816600	1.05371400	-4.83455800
C	-1.64485400	2.66137600	2.28530200
C	-3.11091000	-2.49370500	-1.92615000
C	-2.93734900	2.32528200	-2.36225200
C	-2.05603100	-2.13737400	2.78412800
H	-1.40056100	3.48076700	2.95487700
H	-3.30927300	-3.31553900	-2.60741100
H	-3.10001000	3.03081900	-3.17127600
H	-1.94973100	-2.83694700	3.60757200
O	-0.66082200	0.02535300	-0.32984900
S	-4.66465300	0.07236100	0.99629700
C	-5.75442200	-0.62588800	-0.28012100
H	-5.44323300	-1.65209100	-0.50712700

H	-6.77671400	-0.65165800	0.10808100
H	-5.71098000	-0.03891500	-1.20105200

Supplementary Table 5. Statistics of ligand productive poses in MD simulations. Percent of productive poses for 1000 ns MD simulations with DoxA. Productive pose is defined as a pose where the $O_{Cpd\ I}$-$H_{substrate}$-$C_{substrate}$ angle is >=150° and the $O_{Cpd\ I}$-$H_{substrate}$ distance is <= 5Å. Measurements occurred every 0.5 ns (2000 measurements in total). Simulations were conducted in triplicate.

	**Replicate 1**	**Replicate 2**	**Replicate 3**	**Average**	**Standard Deviation**
			**DOD**		
**C13 %**	55.6	56.7	17	43.1	22.6
**C14 %**	0	0	0.44	0.1	0.3
			**DAU**		
**C14 %**	15.05	4.45	8.5	9.3	5.3

Supplementary Table 6. NMR data for ε-rhodomycinone

	^{13}C	^{1}H
**Position**	**δ ppm**	**δ ppm, *J* Hz**
1	119.7	7.88 (dd, 1.1, 7.5)
2	137.2	7.72 (dd, 7.5, 8.4)
3	124.9	7.32 (dd, 1.1, 8.4)
4	162.7	
4-OH		12.11 (s)
4a	116.0	
5	191.0	
5a	111.4	
6	157.0	
6-OH		12.93 (s)
6a	134.9	
7	62.7	5.35 (dd, 1.4, 5.2)
7-OH		3.48 (ls)
8	34.4	2.29 (dd, 1.4, 14.9) 2.27 (dd, 5.2, 14.9)
9	71.4	
9-OH		3.79 (ls)
10	51.6	4.27 (s)
10a	137.4	
11	155.9	
11-OH		13.45 (s)
11a	111.5	
12	186.2	
12a	133.5	
13	32.6	1.55 (dq, 7.4, 14.0) 1.80 (dq, 7.4, 14.0)
14	6.7	1.15 (t, 7.4)
15	171.3	
16	52.5	3.73 (s)

Supplementary Table 7. Sequences of oligonucleotides used in the work

**RT-qPCR**		
**Gene**	**Forward**	**Reverse**
fdx4<br>*S. peucetius* ATCC 27952/<br>Δ*sufR* mutant	GACTGCCAGATCGAGTGCT	GATCTTGACGGGGTATACGGG
hrdB<br>*S. peucetius* ATCC 27952/<br>Δ*sufR* mutant	GAGTCCGAGTCTGTGATGGC	GAGTCCGAGTCTGTGATGGC
**Analysis of the *fdx4* knockout strain**		
**Primer name**	**Forward**	**Reverse**
F1	GCATGGGCTTCTTGTCGAAG	
R2		TCGTCTCCGAGAACGTCGTG
R3		ATCTTGCCGAGTTGATGGCA

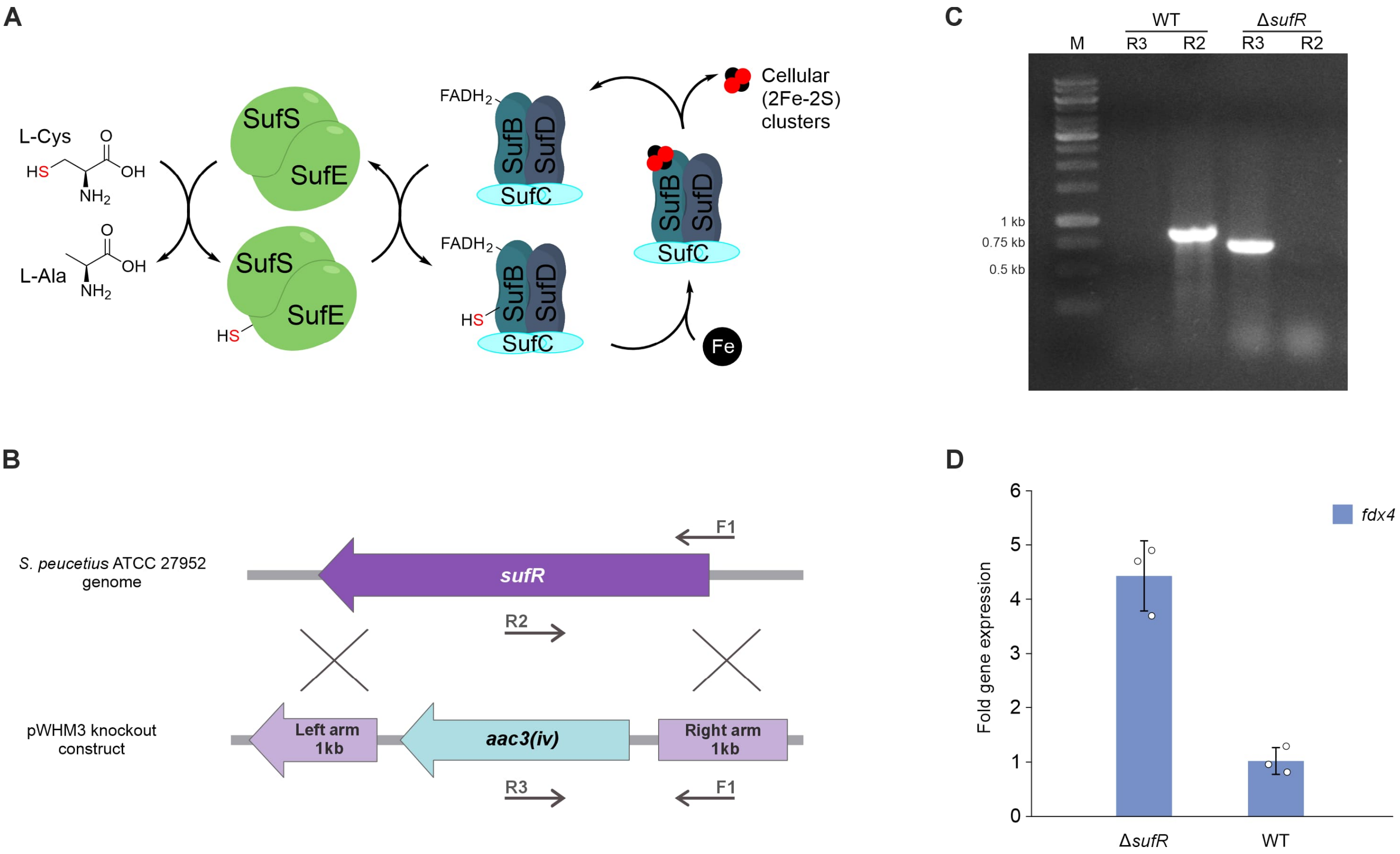

Supplementary Figure 1. Activation of the suf pathway for iron-sulfur cluster biosynthesis in S. peucetius ATCC 27952. A) Proposed functions of the SufB, SufD, SufE and SufS proteins and model for the biosynthesis of iron-sulfur clusters. B) Schematic representation of *sufR* gene disruption in *S. peucetius* ATCC 27952 and the position of PCR primers. C) PCR amplification with two primer sets: the knockout-specific primers F1 and R3 resulted in the amplification of a single ≈ 700 bp fragment in Δ*sufR* mutant, while the use of wild-type-specific primers F1 and R2 in the mutant did not produce a PCR product, indicating successful gene inactivation. The wild-type-specific primers F1 and R2 resulted in the amplification of a single ≈ 800 bp fragment in WT. D) Fold-change in mRNA expression of the *fdx4* gene in *S. peucetius* ATCC 27952 and its Δ*sufR* mutant. Error bars indicate the standard deviation of three technical replicates.

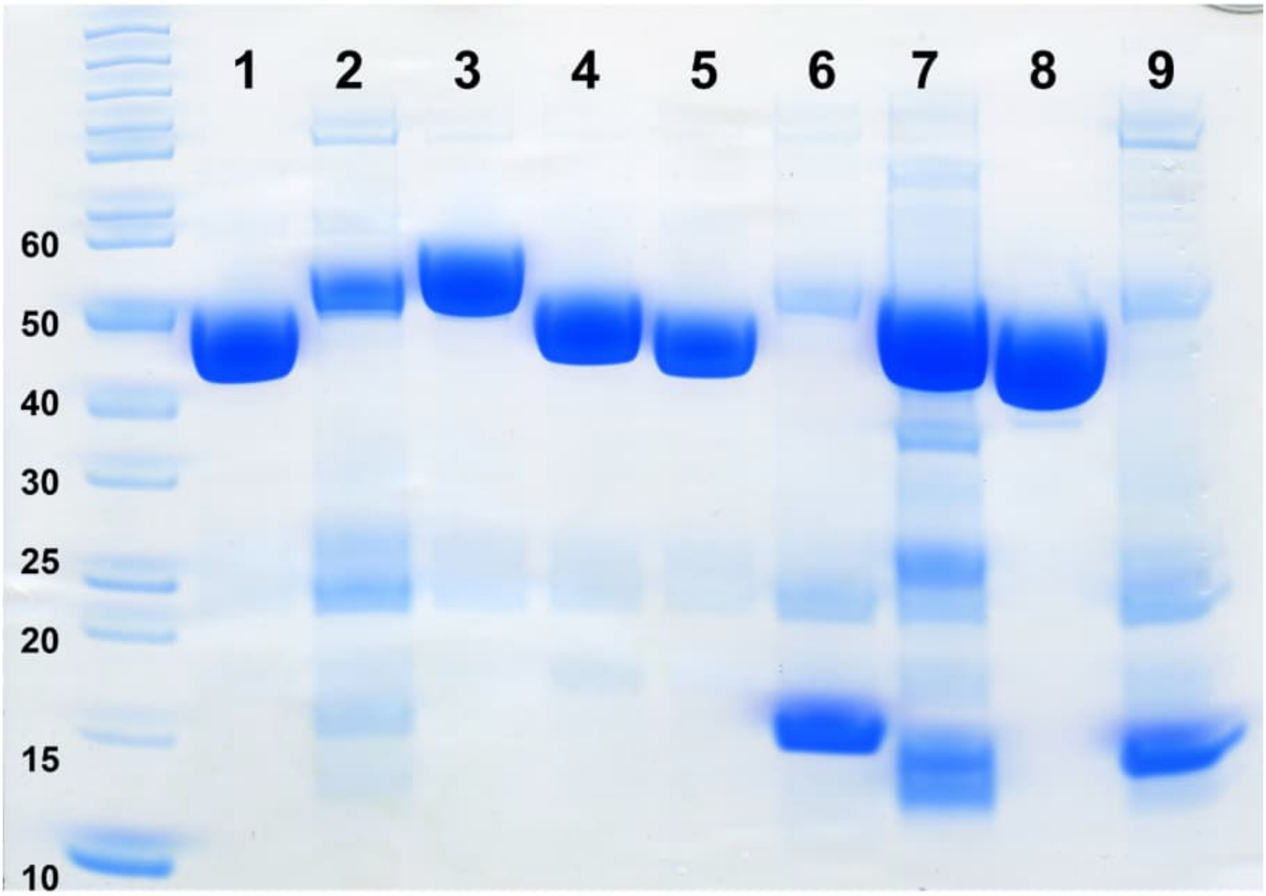

Supplementary Figure 2. Analysis of proteins used in this study by SDS-PAGE. The samples are in the order: Lane 1., SpFdR1 (expected size 46.4 kDa); Lane 2., SpFdR2 (50.1 kDa); Lane 3., SpFdR3 (51.2 kDa); Lane 4., SpFdR4 (47.2 kDa); Lane 5., SpFdR7 (45.0 kDa); Lane 6., SpFdx4 (12.8 kDa); Lane 7., DoxA (48.0 kDa); Lane 8., PdR (47.2 kDa), Lane 9., Pdx(12.9 kDa).

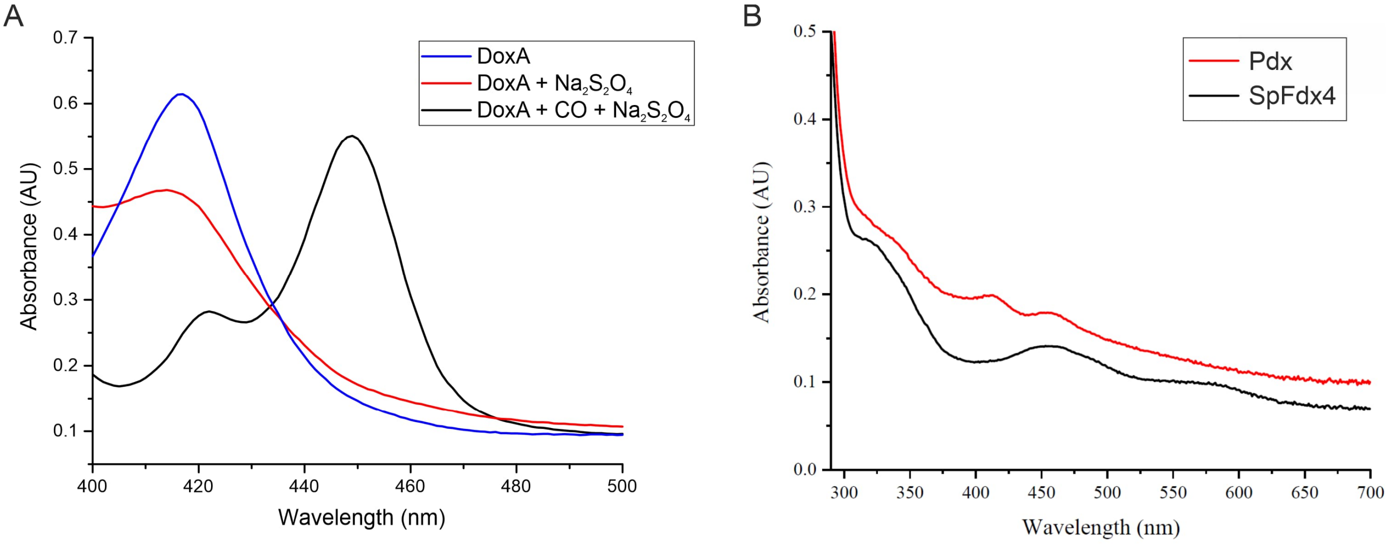

Supplementary Figure 3. Spectroscopic analysis of proteins used in this study. A) Analysis of the haem of DoxA (blue) was investigated by initial reduction using sodium dithionite (red), followed by reaction with carbon monoxide (black), which resulted in the characteristic bathochromic shift from 420 nm to 450 nm. B) Comparison of the UV/Vis spectrum of Pdx (red) and SpFdx4 (black). The UV/vis spectrum of purified SpFdx4 shows peaks at 458 nm and 325 nm, as well as a shoulder at 560 nm, aligning with reported spectra of oxidized Rieske-type ferredoxins. In contrast, Pdx exhibits peaks at approximately 330, 420, and 450 nm expected for putidaredoxin.

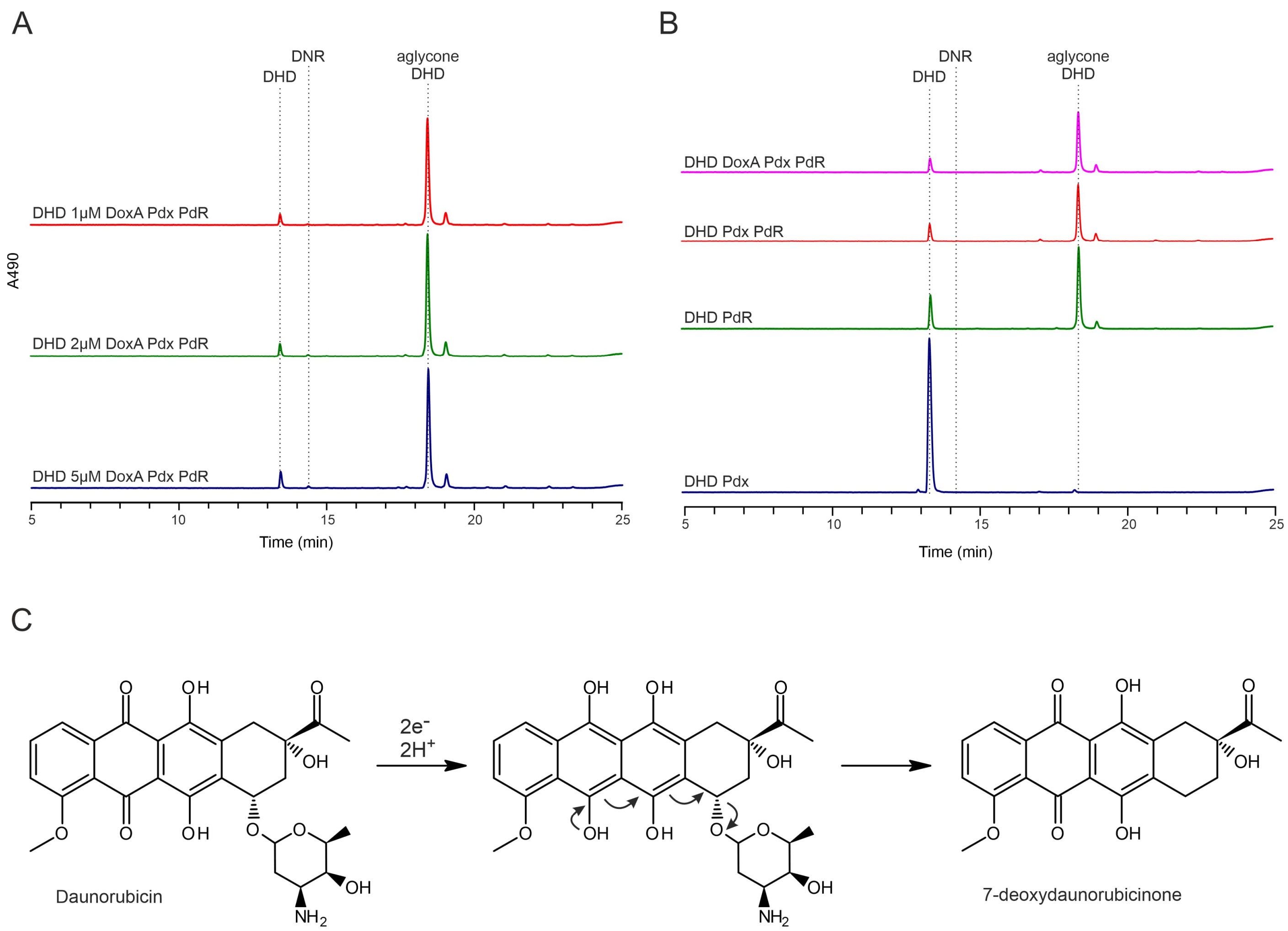

Supplementary Figure 4. Reductive deglycosylation of anthracyclines by ferredoxin reductases. A) Anthracycline deglycosylation products are observed under non-optimal conditions. Reaction with 10 µM Pdx, 5 µM PdR, 1 - 5 µM DoxA, and DHD are shown. B) Anthracycline deglycosylation is catalyzed by ferredoxin reductases even in the absence of ferredoxin and P450 enzymes. Reactions with different combinations of 10 µM Pdx, 5 µM PdR, 1 µM DoxA, and DHD are shown. C) The mechanism of reductive anthracycline deglycosylation is likely to occur via a quinone methide intermediate.

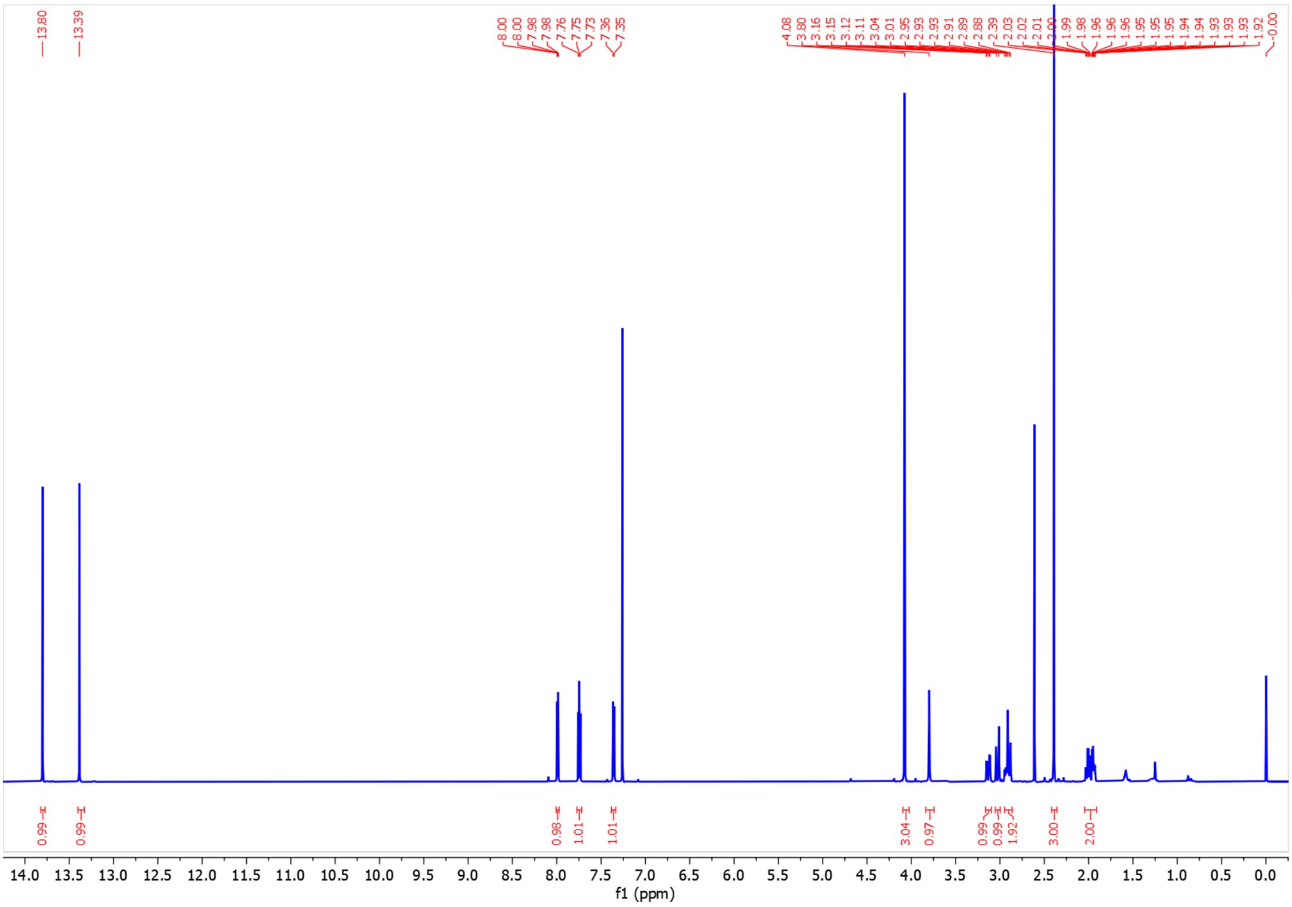

Supplementary Figure 5. ^{1}H NMR spectrum ($CDCl_3$, 600 MHz) of 7-deoxydaunorubicinone

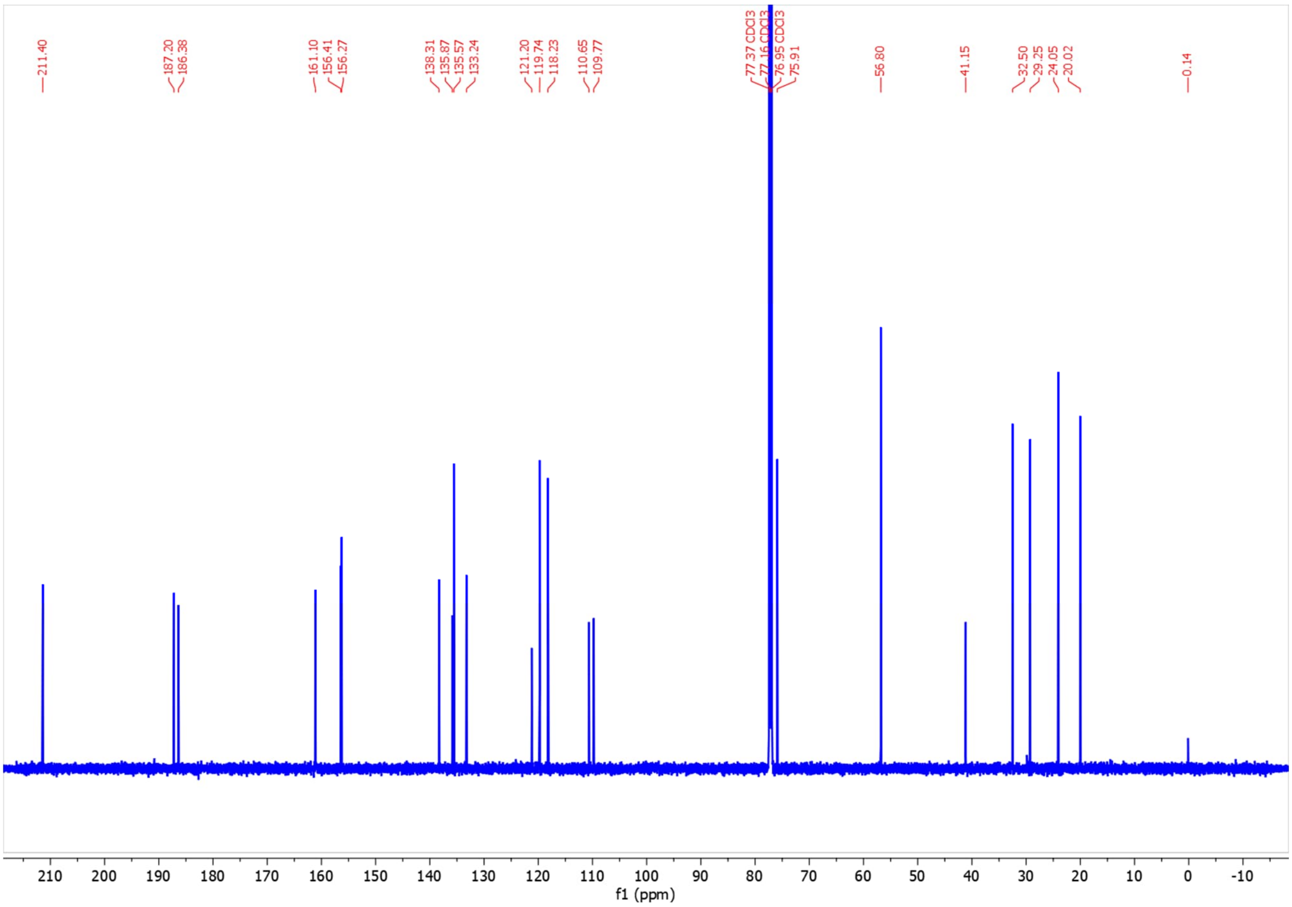

Supplementary Figure 6. ^{13}C NMR spectrum ($CDCl_3$, 151 MHz) of 7-deoxydaunorubicinone

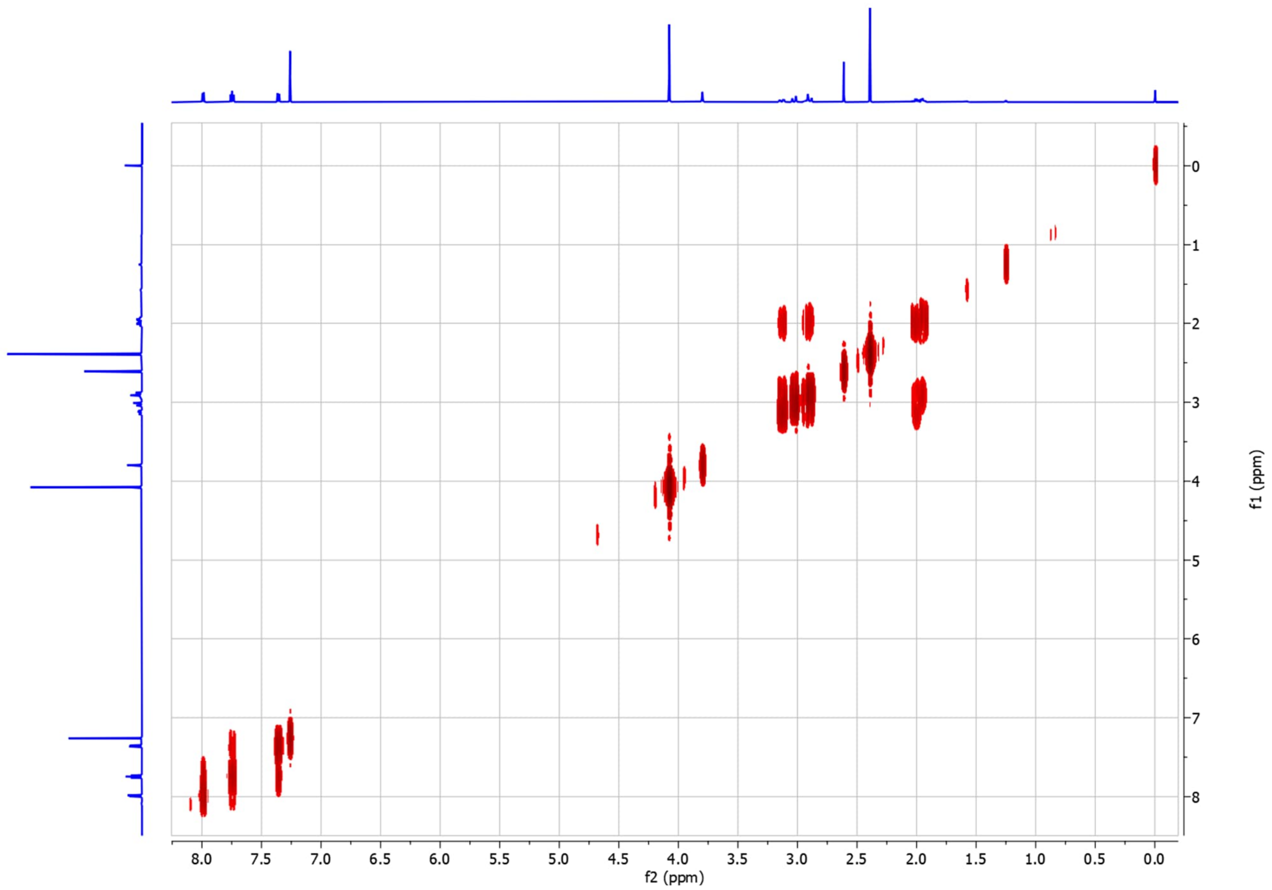

Supplementary Figure 7. COSY NMR spectrum ($CDCl_3$, 600 MHz) of 7-deoxydaunorubicinone

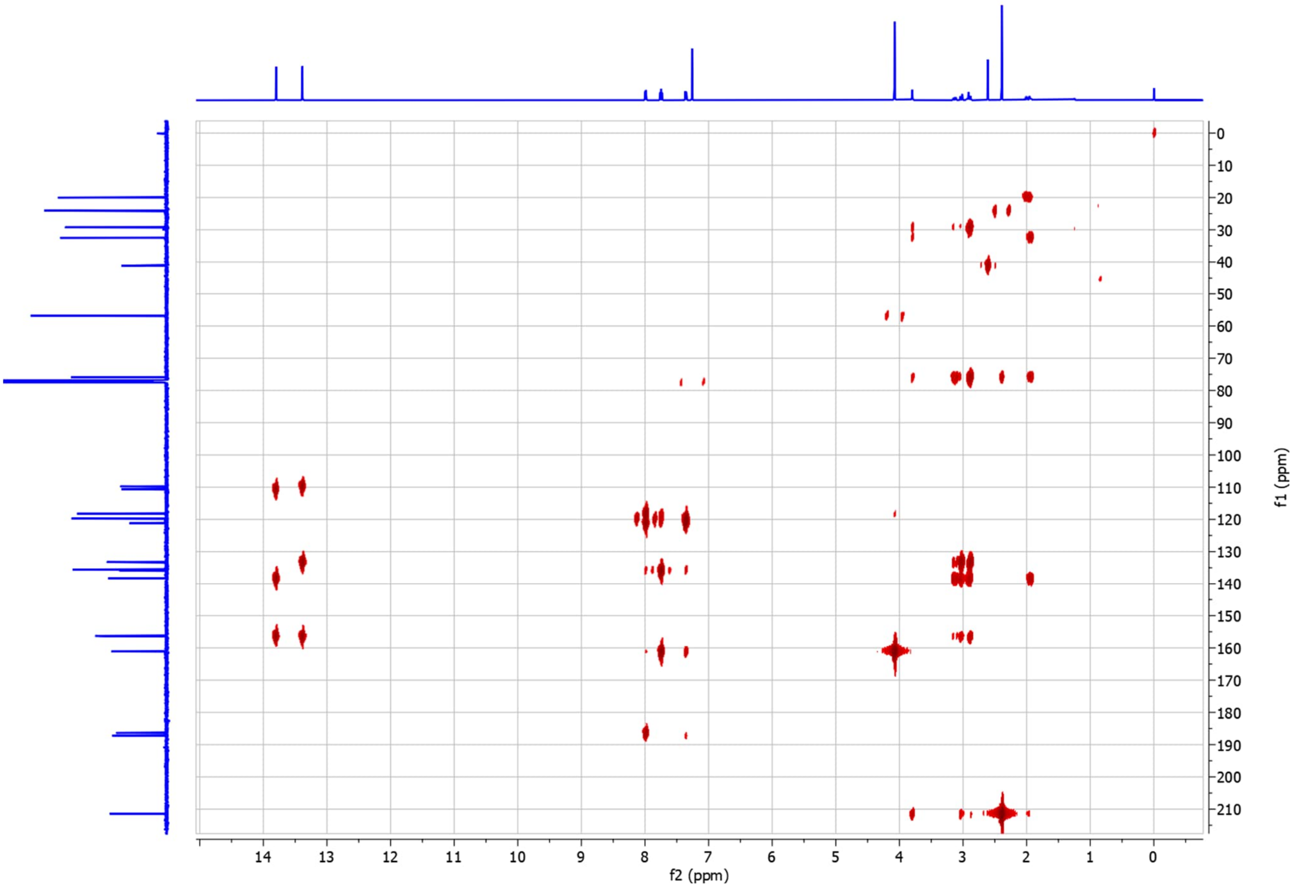

Supplementary Figure 8. HMBC NMR spectrum ($CDCl_3$, 600 MHz) of 7-deoxydaunorubicinone

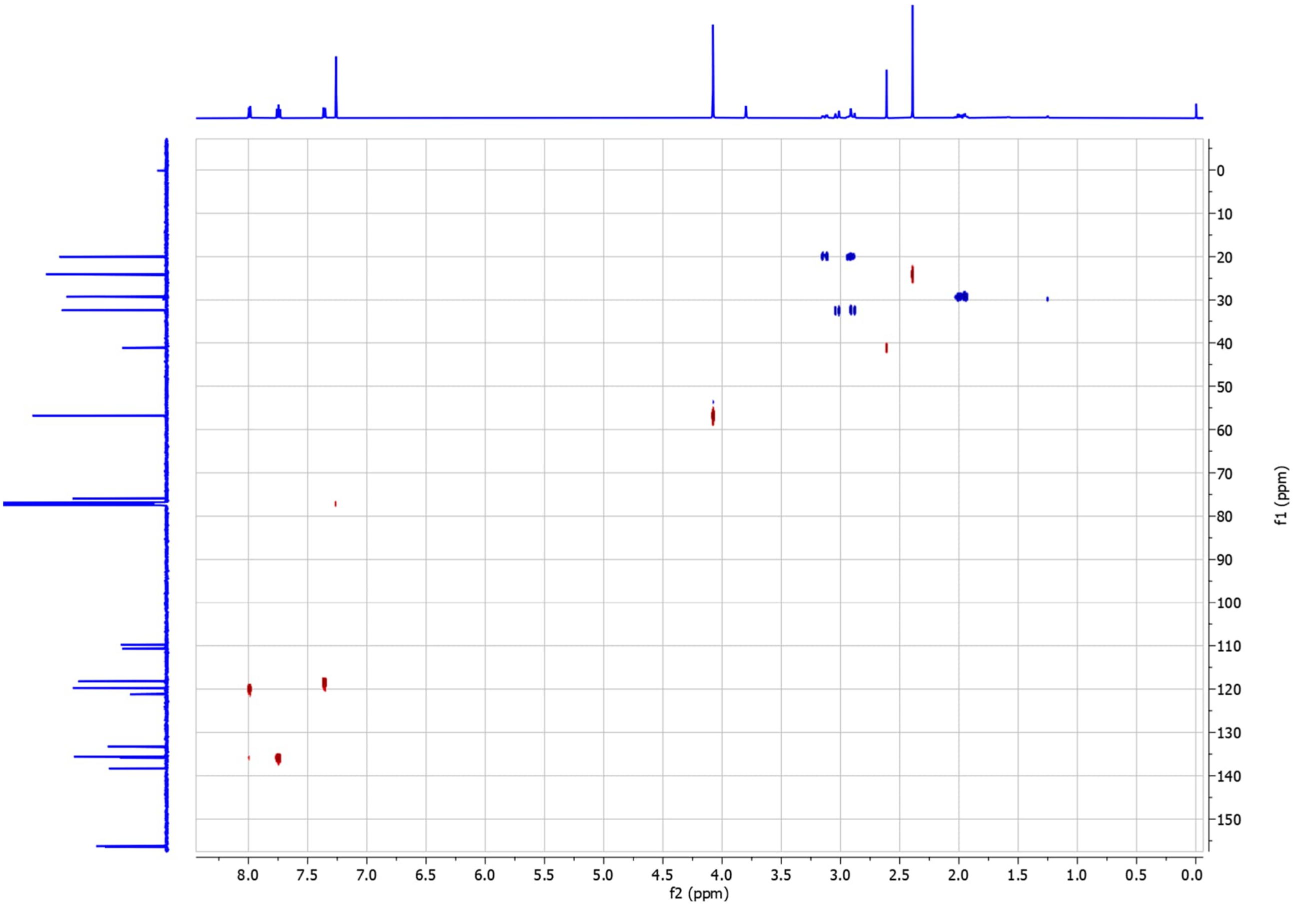

Supplementary Figure 9. HSQC NMR spectrum ($CDCl_3$, 600 MHz) of 7-deoxydaunorubicinone

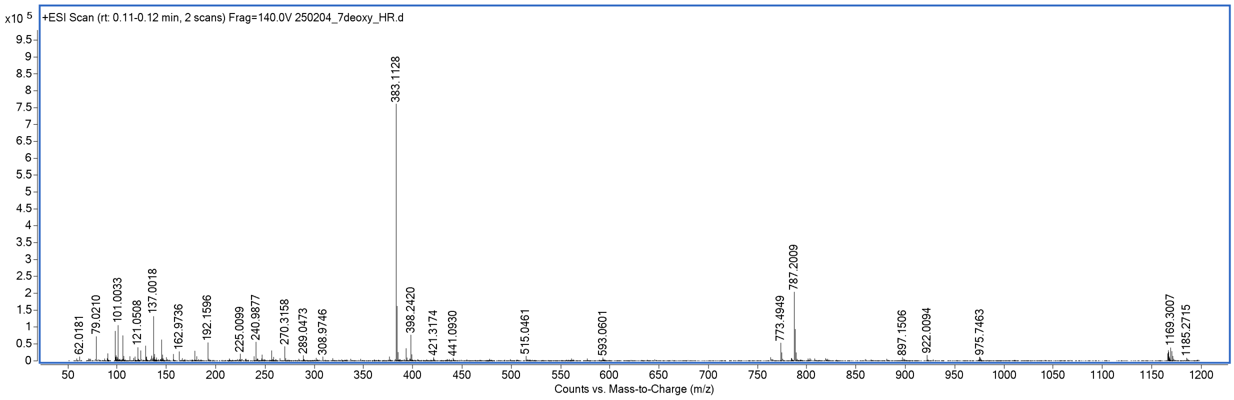

Supplementary Figure 10. HR-MS spectrum of 7-deoxydaunorubicinone. [M+H]+ calc. 383.1125, obs. 383.1128.

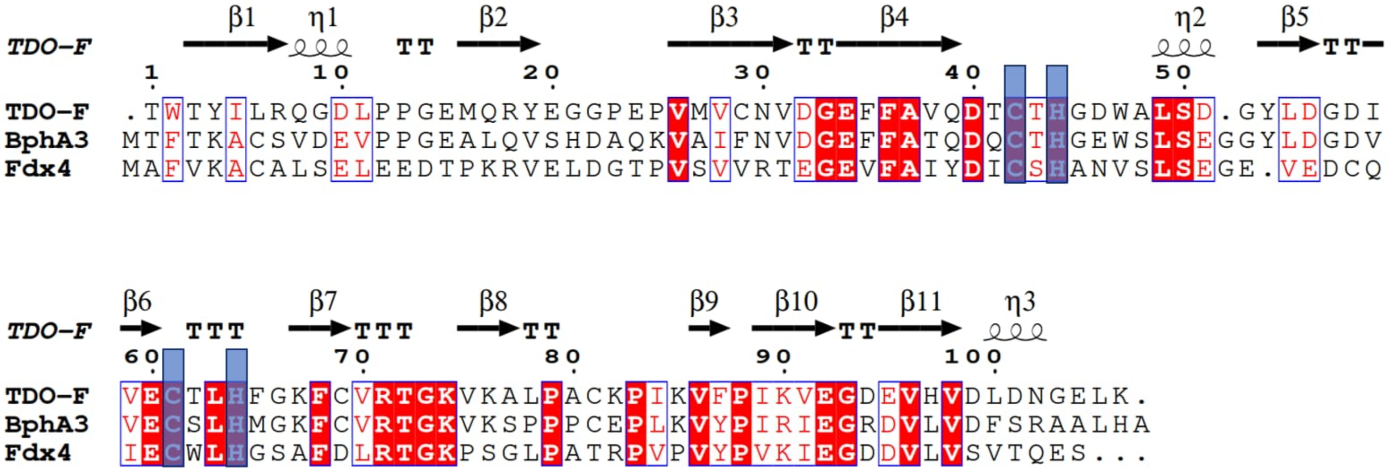

Supplementary Figure 11. Identification of SpFdx4 as a Rieske-type ferredoxin. Structure-based sequence analysis reveals similarity of SpFdx4 to known Rieske-type ferredoxins BphA3 and TDO-F. Histidine and cysteine residues co-ordinating to the [2Fe—2S] cluster are highlighted in blue.

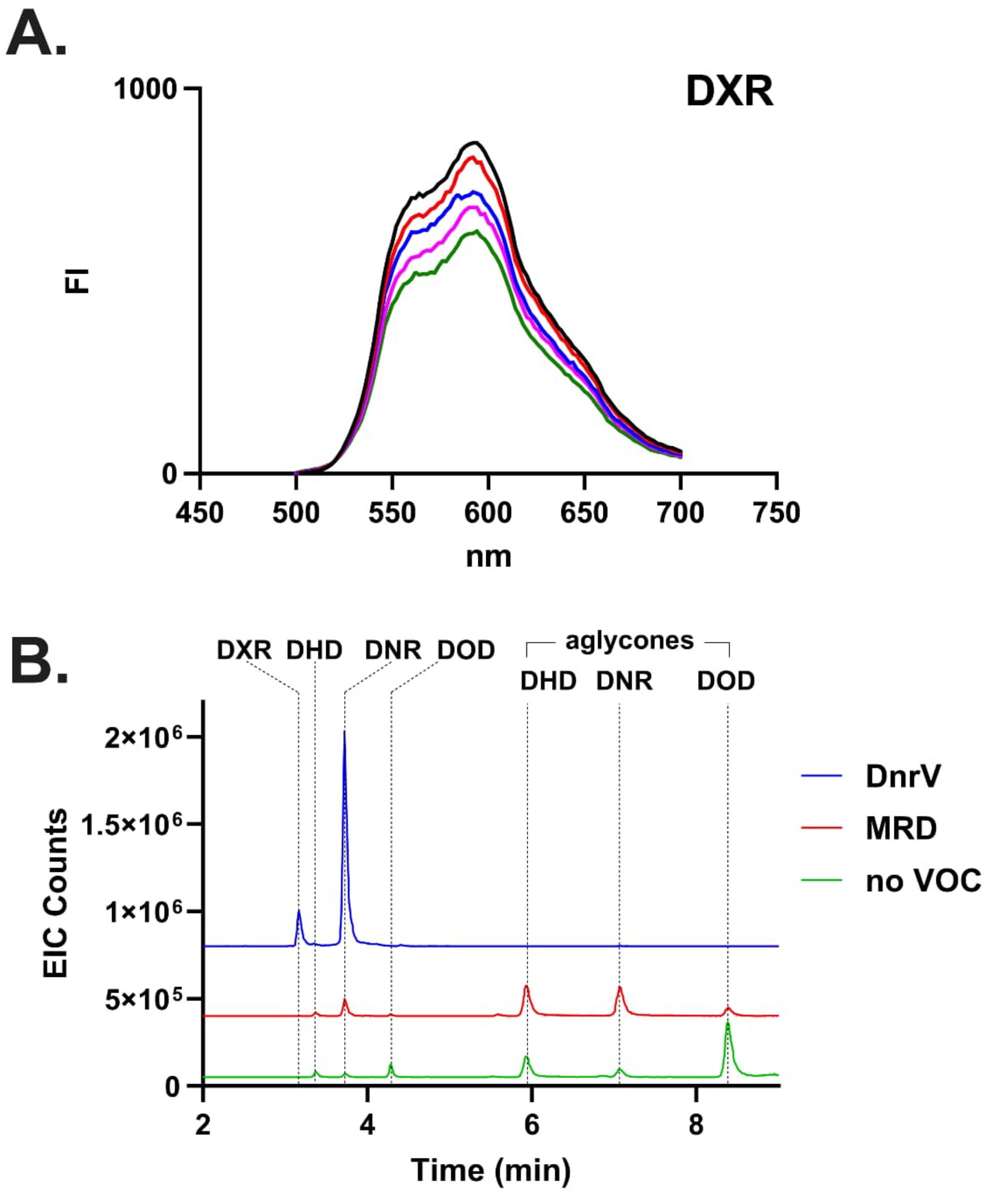

Supplementary Figure 12. **MRD does not interact with anthracyclines or prevent reductive deglycosylation.** A) Fluorescence titration of MRD with DXR. B) DoxA functional assay with 10 μM DoxA, 100 μM DOD, 20 μM Fdx4, 0.1 μM SFR, 0.5 U/μL catalase, 0.5 mM NADP+, 5 mM G6P, 1U/μL G6Pdh, 100 mM phosphate buffer pH 7.4.

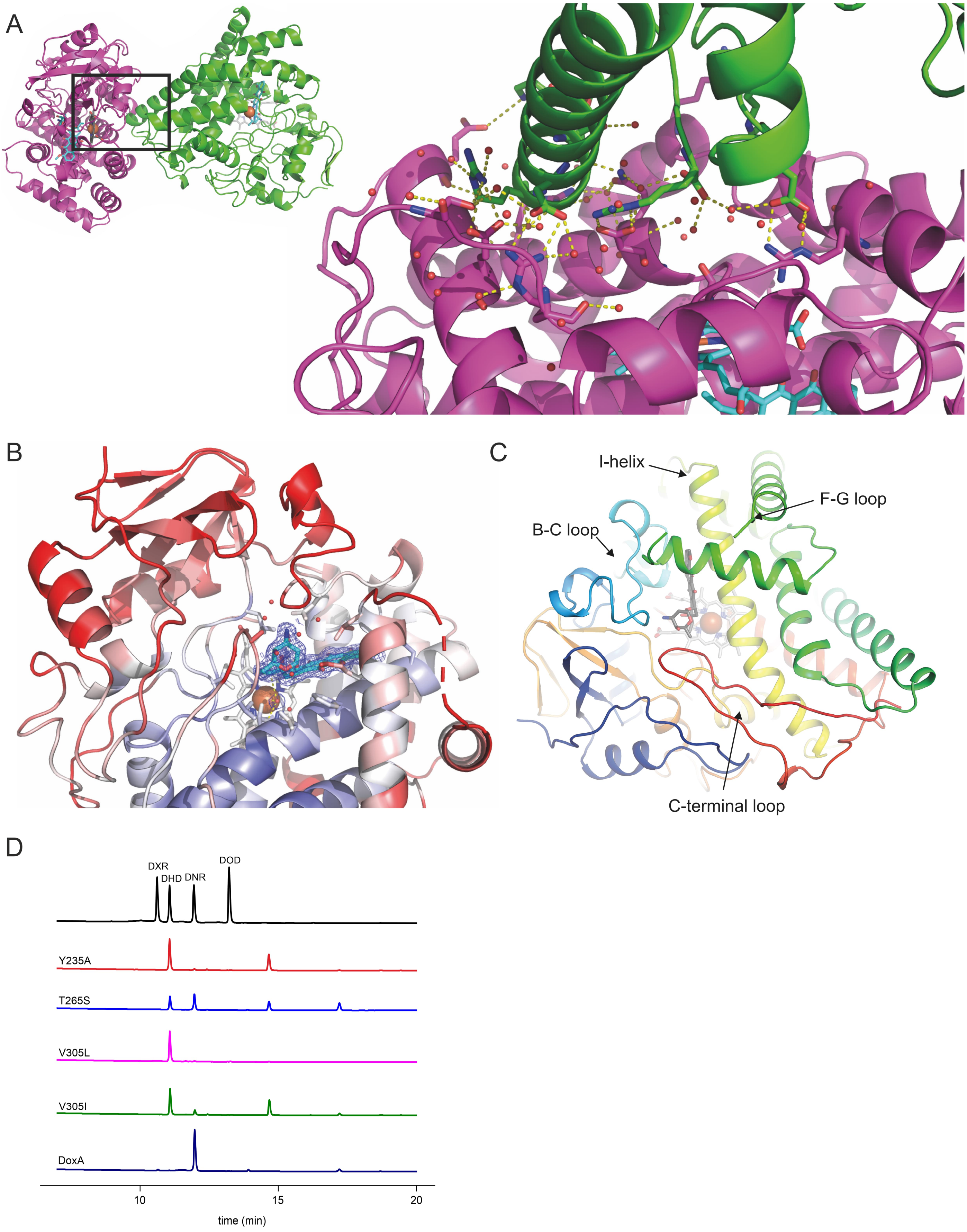

Supplementary Figure 13. Structural and functional analysis of DoxA. A) Zoom of dimer interface shows an intricate hydrogen bonding network close to the heme group, alluding to a possible interaction site for electron

donors. B) Left panel: cartoon representation of DOD-bound DoxA, focussed on the active site around the substrate with B-factors indicated by blue to red colouring. Right panel: similar angle image of the apo structure (density of DOD remains for clarity) indicates highly unstructured regions. C) Putty representation of DOD-bound DoxA complex structure. The protein is colored rainbow from N- to C-terminus and nomenclature for essential loops and helices is indicated. D) Chromatogram traces of DoxA mutants using DOD as a substrate show detrimental effects on enzymatic activity. DoxA mutants Y235A, V305L and V305I converted the substrate to DHD, while T265S harbored higher enzymatic activity with formation of 40% DHD and 60% DNR. The control reaction with wild-type DoxA converted the substrate fully to DNR. Note that the mutations increased the formation of the deglycosylated shunt product 7-deoxydaunorubicinone at 14.5 min.

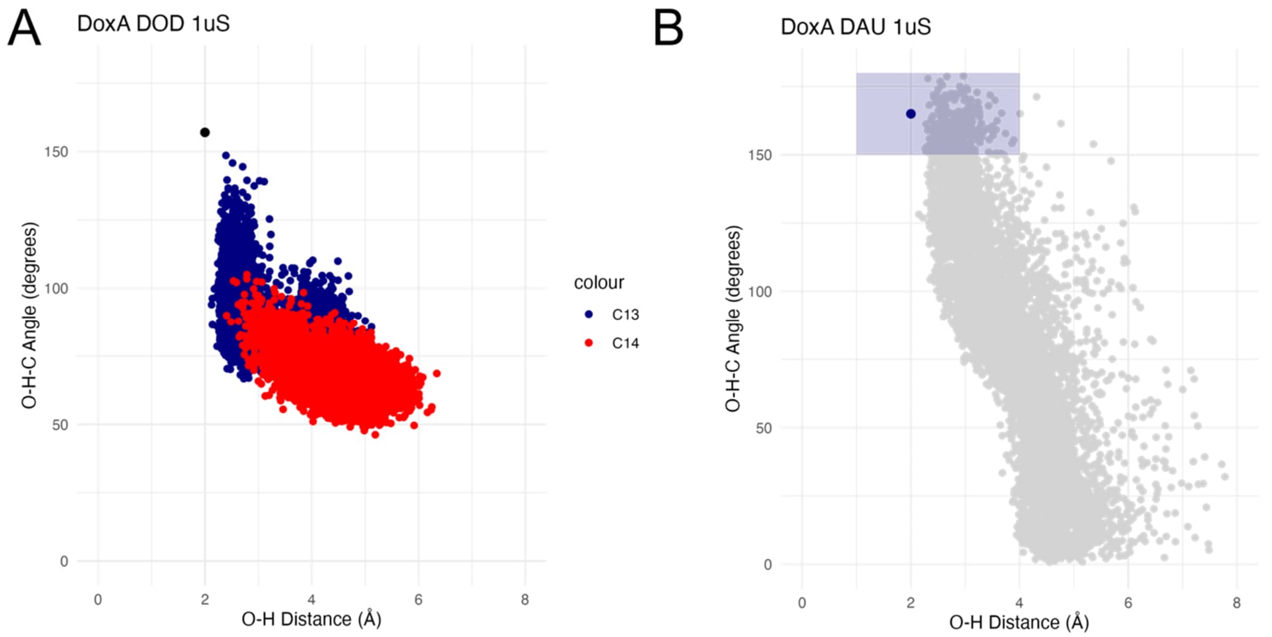

Supplementary Figure 14. Molecular dynamics simulations of DoxA with DOD and DNR. Distance and angle plots for hydrogen atom transfer (HAT) from 1 μs simulations of DoxA substrate complexes. A) Comparison of C13 (blue) and C14 (red) hydrogen atoms on bound DOD substrate. Black circle indicates calculated near attack conformation (NAC) based on DFT analysis. B) Plot of C14 hydrogen atoms (gray) on bound DNR substrate. Black circle indicates estimated NAC based on the DOD calculations and the shaded box indicates selection criteria for a reactive pose.

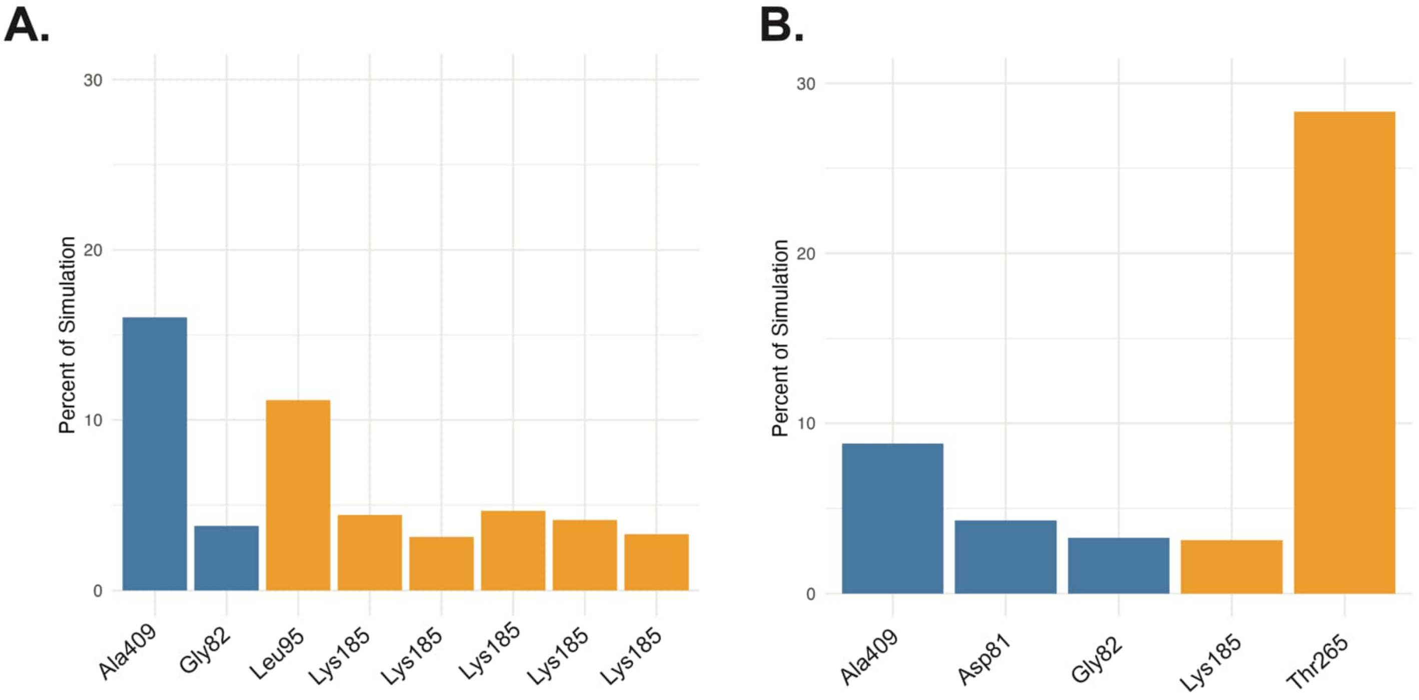

Supplementary Figure 15. Interaction of amino acid residues with ligands in MD simulations.
Hydrogen bond contacts with A) DOD and B) DAU. Data is shown as average of 1uS simulations performed in triplicate. Blue: hydrogen bond acceptor, orange: hydrogen bond donor. Hydrogen bond contact is defined by distance of <= 3Å and angle >= 135°. Only contacts present within 3% or more of simulation are shown.

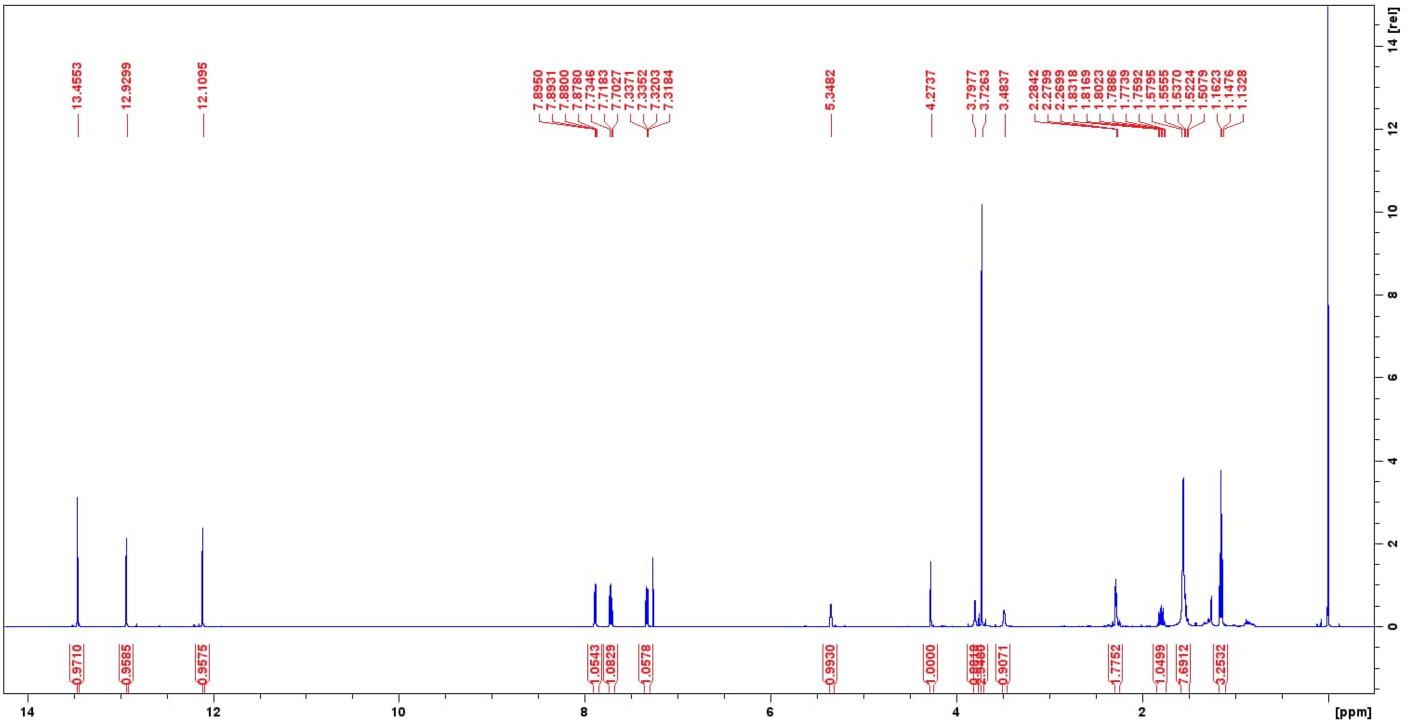

Supplementary Figure 16. ¹H NMR spectrum (CDCl₃, 500 MHz) of ε-rhodomycinone

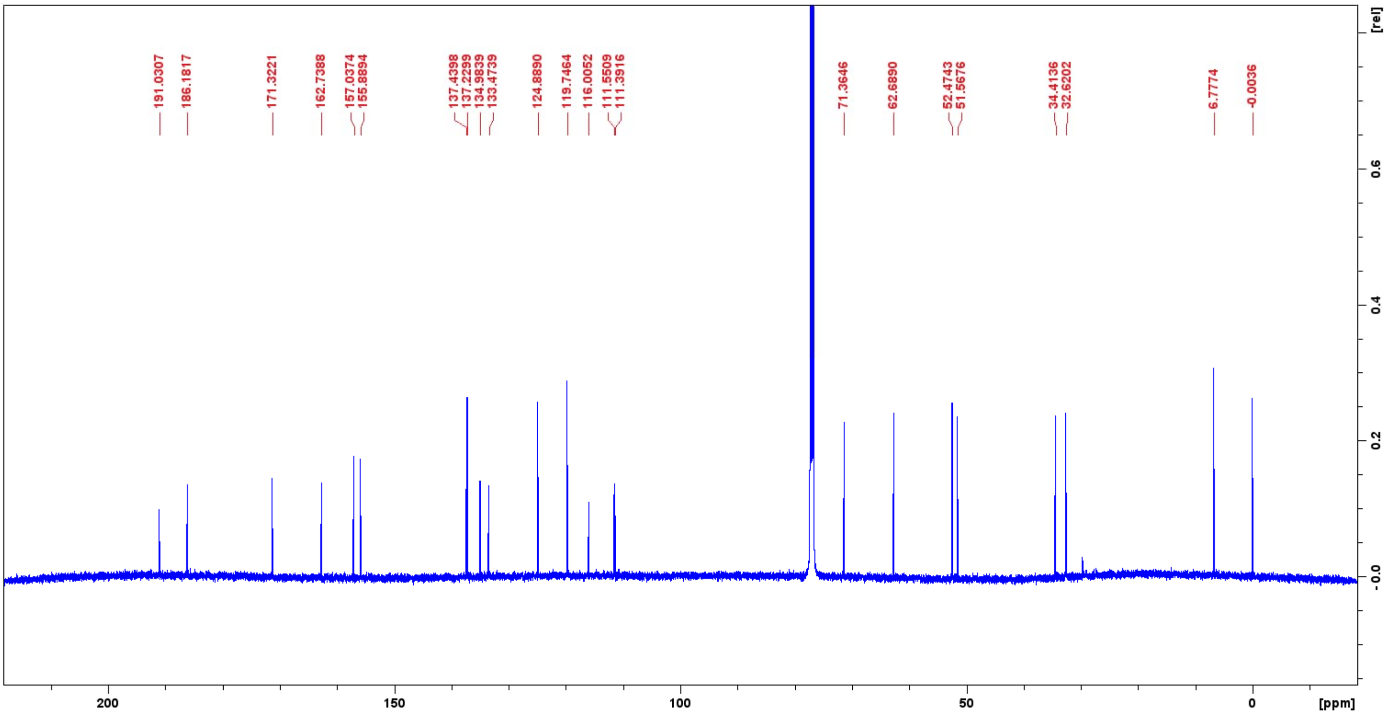

Supplementary Figure 17. ^{13}C NMR spectrum ($CDCl_3$, 125 MHz) of ε-rhodomycinone.

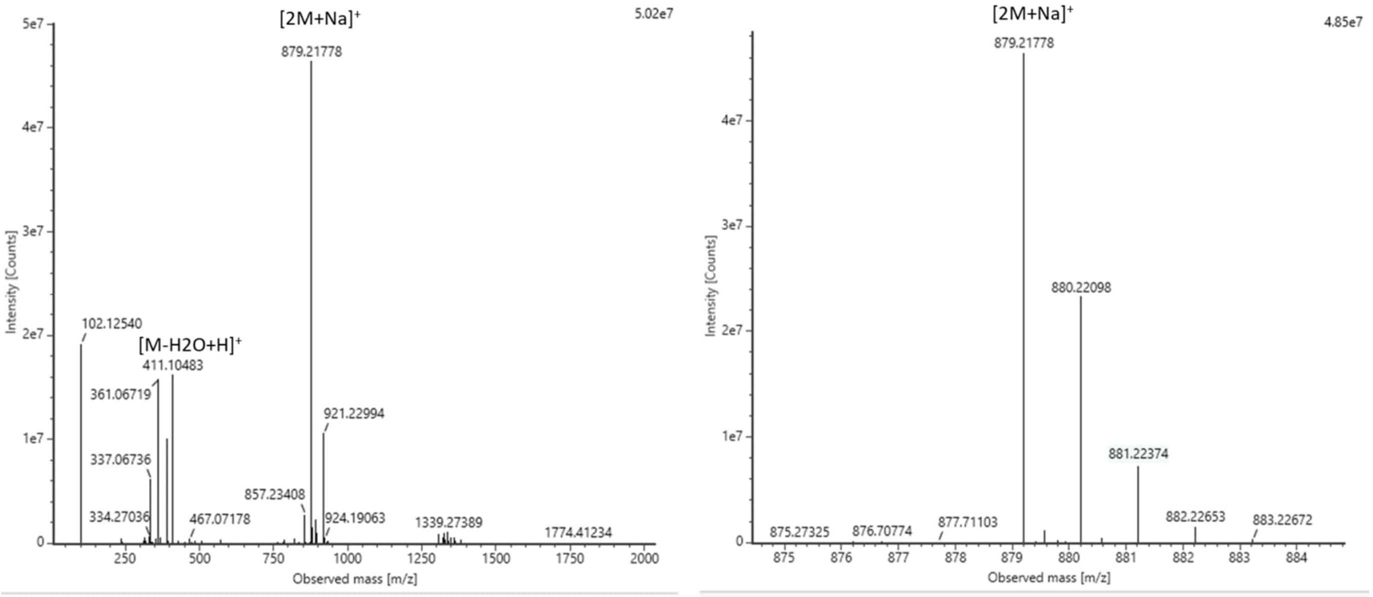

Supplementary Figure 18. HR-MS spectrum of ε-rhodomycinone. $[2M+Na]^+$ calc. 879.2106, obs. 879.2178

Uncropped Scans for Supplementary Figures

Supplementary Figure 1C

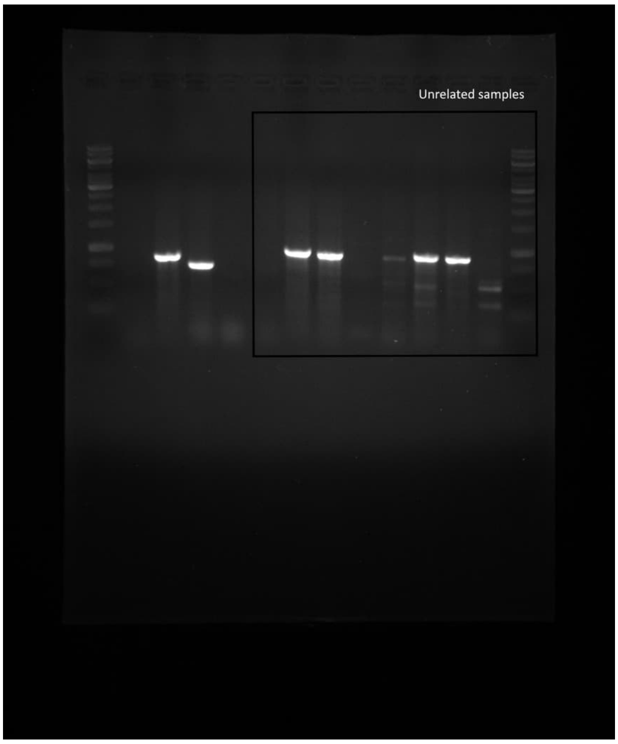

Supplementary Figure 2

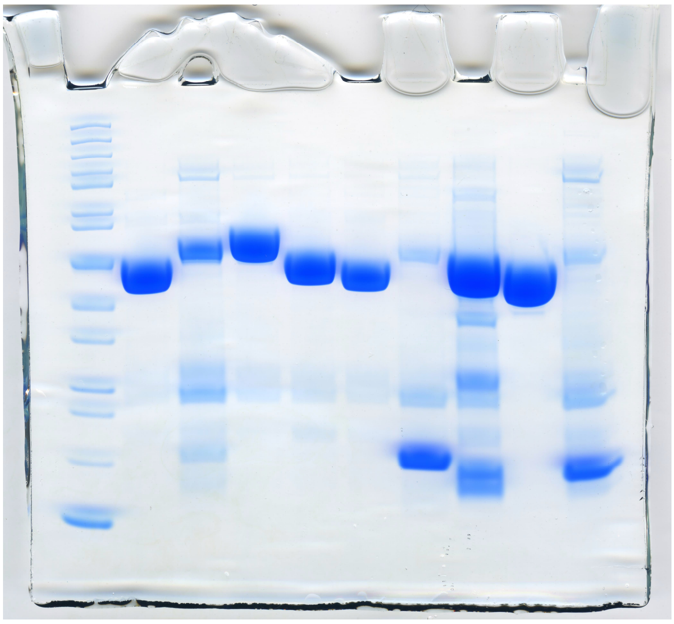

Supplement: Supplementary file 1 — Supplementary Information [file 41467_2026_69194_MOESM1_ESM.pdf]
